# Sugar or Fat? Renal Tubular Metabolism Reviewed in Health and Disease

**DOI:** 10.3390/nu13051580

**Published:** 2021-05-09

**Authors:** Leslie S. Gewin

**Affiliations:** 1 Division of Nephrology and Hypertension, Department of Medicine, Vanderbilt University Medical Center (VUMC), Nashville, TN 37232, USA; l.gewin@vumc.org; 2 Department of Medicine, Veterans Affairs Hospital, Tennessee Valley Healthcare System, Nashville, TN 37212, USA; 3 Department of Cell and Developmental Biology, Vanderbilt University, Nashville, TN 37212, USA

**Keywords:** proximal tubule, acute kidney injury, chronic kidney disease, fatty acid oxidation, kidney injury, kidney metabolism

## Abstract

The kidney is a highly metabolically active organ that relies on specialized epithelial cells comprising the renal tubules to reabsorb most of the filtered water and solutes. Most of this reabsorption is mediated by the proximal tubules, and high amounts of energy are needed to facilitate solute movement. Thus, proximal tubules use fatty acid oxidation, which generates more adenosine triphosphate (ATP) than glucose metabolism, as its preferred metabolic pathway. After kidney injury, metabolism is altered, leading to decreased fatty acid oxidation and increased lactic acid generation. This review discusses how metabolism differs between the proximal and more distal tubular segments of the healthy nephron. In addition, metabolic changes in acute kidney injury and chronic kidney disease are discussed, as well as how these changes in metabolism may impact tubule repair and chronic kidney disease progression.

## 1. Introduction

The kidney receives 25% of the cardiac output and filters approximately 180 L of water daily, while only excreting 1 to 2 L. In addition, over 1.6 kg of salt is filtered but only 3–20 g is eliminated, and significant energy is expended to conserve such a large fraction of water and salt in addition to glucose and other filtered solutes [1]. The human kidney is a complex organ with approximately 1 million nephrons, with each comprising glomerulus and subsequent tubular segments. The tubular compartment of the kidney consists of the proximal tubule (with S1 segment closest to the glomerulus, S2, and S3), loop of Henle, distal convoluted tubule, and the collecting duct. The renal tubules are responsible for conserving 99% of the water and solutes in the glomerular filtrate, as well as maintaining acid/base balance. The metabolic needs and substrate preference (e.g., glucose and fatty acids) vary depending upon the specific tubule segment.

The kidney can be injured acutely by ischemia, toxins, drugs, and infection or can be chronically impaired by diabetes, hypertension, glomerulonephritides, or severe acute kidney injuries. The renal tubules, particularly the proximal tubules, are vulnerable to both acute kidney (AKI) and chronic kidney disease (CKD). There is strong evidence that renal tubular metabolism is altered in both AKI and CKD. Emerging evidence suggests that correcting some of these metabolic changes may attenuate injury or improve recovery, but questions persist about which metabolic perturbations are adaptive versus maladaptive. This review will discuss what is known about metabolism in the healthy kidney tubules, how tubular metabolism changes in kidney injury, and the unanswered questions regarding how metabolic changes may affect tubular repair and tubulointerstitial fibrosis progression, the hallmark of CKD.

## 2. Metabolism in the Healthy Kidney

### 2.1. Fatty Acid Oxidation

**Figure 1:**
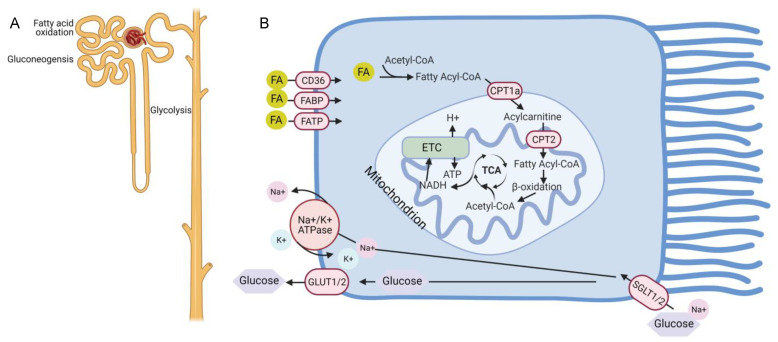
Metabolism in the uninjured nephron and proximal tubule. (**A**) The proximal tubule segment has gluconeogenic capacity and preferentially uses fatty acid oxidation to generate ATP. By contrast, the distal tubules do not have gluconeogenic potential but are better equipped to generate ATP through glycolysis. (**B**) Schematic of metabolism within the proximal tubule showing that glucose is taken up on the apical side by SGLT1/2 transporters and released on the basal side through GLUT1/2. Fatty acids (FA) cross the plasma membrane through CD36, fatty acid binding proteins (FABP), and fatty acid transport proteins (FATP), convert into acetyl-CoA and are transported into the mitochondria through the carnitine shuttle involving the carnitine palmityol-transferases CPT1a and CPT2. Beta oxidation of fatty acyl-CoA produces acetyl-CoA which enters the TCA (tricarboxylic acid) cycle. Oxidation of acetyl-CoA by the TCA produces NADH which enters the electron transport chain (ETC) to generate ATP. Created with BioRen-der.com.

The primary cells responsible for the large reabsorptive capacity of the kidney are proximal tubules which reclaim about 70% of filtered solutes and water. To facilitate this massive amount of water and solute transport, a high number of ATP-consuming transporters are needed. Abundant mitochondria are present in proximal tubules to generate the necessary ATP. Similar to the metabolically active cardiomyocyte, the proximal tubule relies on fatty acid oxidation (FAO) because this fuel source provides 106 ATP units compared to 36 from glucose metabolism (Figure 1A) [2]. Most of the outer kidney, or cortex, is composed of proximal tubules. Consistent with this large mass of proximal tubules, early studies showed that two-thirds of oxygen consumption in the human kidney comes from fatty acid oxidation [3].

Fatty acids can be taken up by renal tubules primarily through the CD36 receptors expressed on plasma membranes, but also through fatty acid binding proteins (FABPs) and fatty acid transport proteins (FATPs) (Figure 1B) [4,5,6,7]. In addition, they can be produced through fatty acid synthase in the cytosol or by metabolism of phospholipids through phospholipase A2 [2]. Long-chain fatty acids (LCFA) such as palmitate require the carnitine shuttle for transport into mitochondria where oxidation and ATP generation occurs. The carnitine shuttle consists of carnitine palmitoyl-transferase 1 (CPT1), located on the outer mitochondrial membrane, which converts fatty acyl CoA into a long-chain acylcarnitine, allowing movement into the mitochondrial matrix [8]. The carnitine palmitoyltransferase-2 (CPT2) enzyme then reconstitutes acyl-CoA, which undergoes β-oxidation in the mitochondria. The resultant acetyl-CoA enters the tricarboxylic acid (TCA) cycle and becomes oxidized, leading to the reduction of NAD (nicotinamide adenine dinucleotide) and FAD (flavin adenine dinucleotide) to NADH and FADH, respectively. NADH and FADH subsequently enter the electron transport chain (ETC) and provide electrons to generate the electrochemical gradient leading to ATP production.

CPT1 is considered the rate-limiting enzyme in the process of fatty acid oxidation [9]. There are three CPT1 isoforms (a, b, and c), and CPT1a is highly expressed throughout the kidney, liver, and other organs, whereas CPT1b is primarily expressed in the skeletal muscle, heart, and adipose tissues, and CPT1c localizes to the brain and testes [9]. Recent single cell transcriptome studies in the adult mouse and human kidneys have confirmed the predominance of the CPT1a isoform and its ubiquitous renal expression [10,11]. CPT1 is needed for mitochondrial import of LCFA but is not required for medium-chain fatty acids (MCFA). Perfused rat kidneys were able to take up both LCFA (palmitate) and MCFA (octanoate) based upon isotope-labeled studies [12]. Very long chain fatty acids (VLCFA) can be oxidized by peroxisomes but these organelles lack respiratory chain enzymes and, thus, cannot generate ATP [13]. Rather, the products of peroxisomal oxidation can be transported into mitochondria for further oxidization into acetyl-CoA and ATP production via the ETC. The highest density of peroxisomes within the kidney resides in the proximal tubule, suggesting that fatty acid oxidation (FAO) in the proximal tubule may be mediated by both mitochondria and peroxisomes [14,15]. Peroxisomal oxidation of long chain fatty acids such as palmitate can compensate when mitochondrial oxidation is impaired, which has important implications for renal injury [16]. Although all renal tubule segments are able to oxidize fatty acids, the rates appear to be directly related to the mitochondrial content, which is greatest in the proximal tubule segments and the distal convoluted tubule [17,18]. Given the limited ability of the healthy proximal tubule to metabolize glucose as discussed below, FAO is the preferred energy substrate for this tubule segment.

### 2.2. Glucose Metabolism

FAO may be the preferred energy substrate for proximal tubules, but the kidney is an important organ for glucose reabsorption, production, and utilization. Most of the filtered glucose, a total of 180 g per day, is reclaimed by one of two sodium-dependent glucose cotransporters (SGLT) located on the apical surface of the proximal tubule [19,20]. SGLT2 is a low-affinity, high-capacity transporter located primarily in the S1 and S2 segments of the proximal tubule [21,22]. SGLT2 couples the transport of sodium and glucose in a 1:1 ratio and reabsorbs up to 90% of filtered glucose [23]. By contrast, SGLT1 is a high-affinity, low-capacity transporter located in S3 segment of the proximal tubule and transports sodium in a 2:1 ratio with glucose [24,25]. SGLT2 has recently garnered much attention as the target of many drugs (e.g., empagliflozin and dapagliflozin) that have renoprotective and cardioprotective effects even in patients without diabetes [26,27]. The mechanism of SGLT2-mediated protection in CKD and heart failure is beyond the scope of this review but highlights the importance of glucose handling by the kidney.

A large amount of glucose enters the proximal tubules, but as mentioned above, little glucose is metabolized in the uninjured proximal tubules. Instead, facilitative transporters of the GLUT family are located on the basolateral membrane and allow glucose to move down a concentration gradient back into the circulation. GLUT2 is the transporter found in S1 and S2 proximal tubule segments that matches the high-capacity, low-affinity glucose flux initiated by SGLT2 on the apical surface. Similarly, GLUT1 provides an exit pathway for glucose that entered the S3 segment through SGLT1 [28,29]. Thus, the proximal tubule relies primarily on FAO for energy, but a large amount of glucose flux occurs across these cells, and disruption of this glucose movement has been harnessed for therapy of CKD and heart failure.

The kidney and liver are the only two organs capable of releasing glucose into the circulation as other tissues lack glucose 6-phosphatase, required for glucose formation from glucose-6-phosphate. Glucose can be produced by glycogenolysis or gluconeogenesis. Glycogen is broken down into glucose-6-phosphate in glycogenolysis, but the kidney does not have significant glycogen stores [30]. In gluconeogenesis, substrates such as lactate, glycerol, alanine, and glutamine can lead to the production of glucose-6-phosphate. In the kidney, studies suggest that lactate is the predominant precursor for gluconeogenesis [31]. Initial studies measuring renal artery and vein glucose concentrations did not find much net glucose change in the kidney. However, studies using isotopically labeled glucose showed that the kidney both produces and metabolizes considerable amounts of glucose [32]. Similar approaches in humans show that the kidney accounts for approximately 25% of all glucose released into the circulation [33]. In diabetic patients, there is evidence that gluconeogenesis is further upregulated by both the kidney and the liver [34]. These findings suggest that renal gluconeogenesis may contribute to hyperglycemia in diabetic patients. For diabetic patients with chronic kidney disease, this loss of renal gluconeogenic activity likely contributes to hypoglycemic episodes in addition to reduced insulin clearance that results from impaired kidney function.

**Figure 2:**
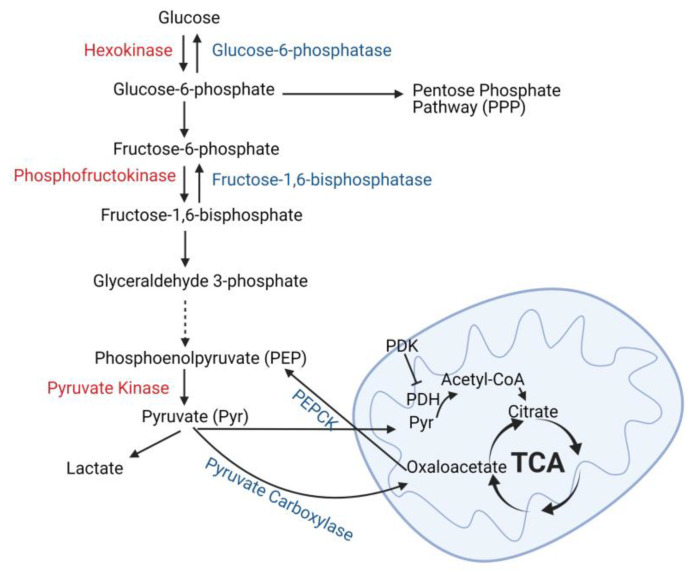
Glucose metabolism and production in the kidney tubules. Glucose is metabolized to glucose-6-phosphate which can enter the pentose phosphate pathway or be metabolized to pyruvate (glycolysis). The key enzymes necessary for glycolysis are listed in red, and these enzymes are predominately expressed in distal tubules of the kidney. Pyruvate can either be converted into lactate (anaerobic glycolysis) or enter the mitochondria where it is converted into acetyl-CoA by pyruvate dehydrogenase (PDH) and oxidized by the tricarboxylic acid (TCA) cycle. Enzymes associated with gluconeogenesis are shown in blue and their expression in the kidney is restricted to proximal tubules. Phosphoenolpyruvate carboxykinase (PEPCK), pyruvate dehydrogenase kinase (PDK). Created with BioRender.com.

The kidney both produces and consumes glucose, but these actions are strictly compartmentalized by specific tubule cell type. Gluconeogenesis is restricted to the proximal tubules which express the key enzymes necessary for this process: glucose-6-phosphatase, phosphoenolpyruvate carboxykinase (PEPCK), and fructose-1, 6-diphosphatase (Figure 2) [35,36,37]. By contrast, utilization of glucose as a metabolic fuel is restricted to the distal tubules in the healthy kidney. Glycolysis is the metabolism of glucose into pyruvate, which can be further oxidized through the TCA cycle or metabolized into lactate. Glycolytic enzymes such as hexokinase, phosphofructokinase, and pyruvate kinase have the highest expression in thick ascending limbs and distal and collecting ducts [38,39,40]. Consistent with the enzyme expression levels, glucose oxidation and ATP generation from glucose in microdissected rat proximal tubules was significantly less than more distal tubule segments [41,42]. Studies have confirmed that distal tubules can metabolize glucose to lactate even in aerobic conditions, and this ability is greatly augmented by antimycin A, which blocks oxidative respiration [43]. By contrast, lactate production from glucose was minimal in microdissected rat proximal tubules, and antimycin A failed to induce a rise in lactate, suggesting that the healthy proximal tubule has a limited ability to metabolize glucose to lactate [43]. Thus, glucose is produced by the proximal tubule through gluconeogenesis, whereas it is metabolized through glycolysis by the distal nephron segments.

### 2.3. Amino Acid Metabolism

Nearly 70 gm of free amino acids per day is filtered by the glomerulus, and its reabsorption from the lumen is predominantly mediated by the proximal tubule [44]. Amino acids are absorbed into renal tubules through a combination of diffusion, facilitated diffusion, and sodium-dependent active transport. Amino acid transporters are highly expressed on the proximal tubule’s brush border lining the tubular lumen, but basolateral amino acid transporters also reabsorb amino acids for specialized functions [45]. Some of these reabsorbed amino acids can be substrates for gluconeogenesis as discussed above. Alternatively, amino acids can enter the TCA cycle at various points and become oxidized. Branched chain amino acids (BCAA) consisting of leucine, valine, and isoleucine are also an important source of energy. BCAA are metabolized by an initial transamination step through branched chain aminotransferases (BCAT) to form branched chain α-ketoacids, which then undergo oxidative decarboxylation by the branched chain alpha-ketoacid dehydrogenase (BCKDH) complex [46]. Products from BCAA metabolism enter the TCA cycle, either as acetyl-CoA or succinyl-CoA, where they undergo oxidation. BCAT and BCKDH are expressed and active within the kidney, and BCAA oxidative flux in the kidney is higher than other tissues except for the heart and brown fat [46]. Approximately 8–13% of human BCAA metabolism is estimated to occur in the kidney [47].

Metabolism of certain amino acids facilitate other biologic functions independent from generation of energy, such as the role of glutamine metabolism in acid/base balance. Glutamine can be metabolized by the proximal tubule into glutamate, which, in turn, is converted into the TCA cycle intermediary α-ketoglutarate. These reactions also generate ammonia, some of which enters the urine through the sodium–hydrogen exchanger-3 (NHE3), and bicarbonate, which is reabsorbed into the circulation through the basolateral sodium-coupled bicarbonate co-transporter, isoform 1A (NBCe-1A) [48]. During acidosis, proximal tubular metabolism of glutamine to produce ammonia and reclaim bicarbonate is upregulated to help maintain acid/base homeostasis. This is achieved both by upregulating glutaminase, the enzyme catalyzing glutamine metabolism, as well as increased expression of basolateral glutamine transporters to augment uptake into the proximal tubule [49]. Metabolism of other amino acids also contributes to ammoniagenesis and bicarbonate reabsorption, but glutamine is the primary source.

The kidney is an important site for the metabolism of other amino acids that play important biological roles. Citrulline, generated by enterocytes of the small intestine, is primarily taken up by the kidneys where it is metabolized to arginine [50]. Arginine is a precursor for nitric oxide (NO), which is important for endothelial function and blood flow regulation among other effects (immune response, protein synthesis). The kidney also converts phenylalanine into tyrosine through the enzyme phenylalanine hydroxylase, expressed in both the kidney and liver [51]. Tyrosine plays an important role in the production of neurotransmitters and thyroid hormones, and the conversion rates of phenylalanine to tyrosine are reduced 50% in patients with end-stage renal disease compared to those with normal renal function [52]. These are not the only examples of the importance of amino acid metabolism by the kidney, which has been more extensively reviewed by others [53]. Though glucose and fatty acids may be more important sources of energy for the healthy kidney, renal amino acid metabolism clearly plays an integral role in the organism’s homeostasis.

## 3. Metabolism of the Injured Kidney

As mentioned earlier, the proximal tubules are the workhorses of solute reabsorption, and abundant mitochondria are necessary to generate the ATP required to support the transporters needed to reclaim the solute load. Production of ATP by the mitochondrial ETC requires oxygen. Therefore, tubule segments, particularly the proximal tubule, have high oxygen requirements that render these cells vulnerable to renal injury. The S3 segment of the proximal tubule is particularly susceptive to injury due to its deep location within the kidney where blood flow and oxygen tensions are lower. Impaired oxygen delivery due to disrupted hemodynamics (e.g., sepsis and cardiopulmonary bypass) is a key feature of many renal injuries and leads to hypoxic renal tubules, with impaired function. The high expression of transporters in the proximal tubule also makes this tubule segment prone to injury by toxins (e.g., mercury, lead, and aristolochic acid), or drugs such as aminoglycosides. Injury can either occur as acute kidney injury (AKI) or chronic kidney disease (CKD) in which the injury is ongoing as in hypertension or diabetes. In clinical practice, patients with AKI are at much greater risk for developing CKD, and those with CKD have a higher chance of having AKI, suggesting that the two processes are interrelated [54]. However, there may be important differences in the nutrient availability of the kidney recovering from AKI compared to that in CKD. Thus, the literature on metabolism in AKI versus CKD will be reviewed separately.

## 4. Renal Tubular Metabolism in AKI

Acutely injured renal tubules can undergo cell death through apoptosis or necrosis or slough off due to reduced adhesion from altered integrin expression [55]. The surrounding epithelial cells de-differentiate, migrate, and proliferate to repair the injured tubule epithelium. How these surviving epithelial cells respond can determine whether the kidney undergoes successful repair or progresses to tubulointerstitial fibrosis.

### 4.1. AKI and Anaerobic Glycolysis

**Figure 3:**
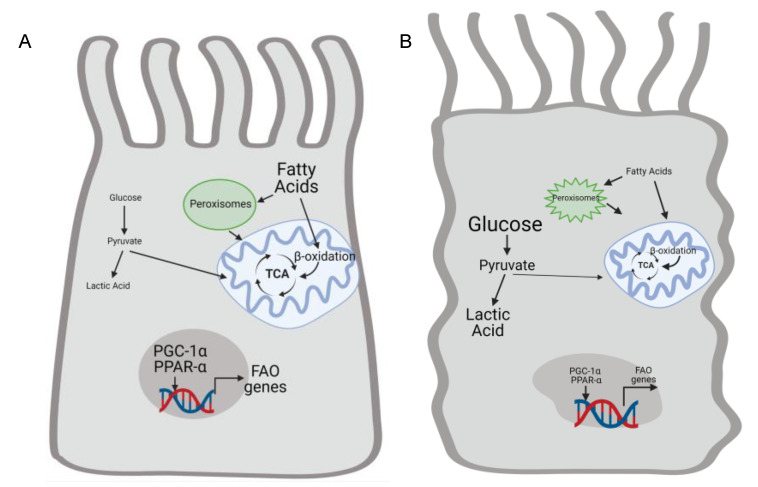
Renal injury alters proximal tubule cell metabolism by suppressing fatty acid oxidation and increasing anaerobic glycolysis. (**A**) Healthy proximal tubule (PT) cells relies utilization fatty acid oxidation by peroxisomes and mitochondria to generate ATP. Transcription factors such as PCG-1α and PPAR-α increase mitochondrial biogenesis and expression of genes related to fatty acid oxidation. Conversel y, glycolysis is not a big source of energy in the uninjured proximal tubule. Kidney injury impairs mitochondrial function and decreases expression of PGC-1α and PPAR-α (**B**). Therefore, fatty acid oxidation declines and injured PT cells rely on glycolysis to help meet energetic demands. Anaerobic glycolysis leads to increased levels of lactic acid. Created by BioRender.com.

There is strong evidence that FAO, the preferred metabolic pathway for generating energy in proximal tubules, is suppressed in AKI in favor of glucose metabolism to lactic acid (Figure 3). Glycolysis technically refers to glucose metabolism to pyruvate, and the term “anaerobic glycolysis” is used here to the indicate metabolism of glucose to pyruvate and then lactic acid as contrasted with glucose oxidation (glucose metabolism to pyruvate which then enters the TCA cycle). Increased glucose uptake and anaerobic glycolysis were also described in rats with mercuric chloride–induced AKI [56]. Ischemia reperfusion injury (IRI) increased levels of lactate and pyruvate in the injured kidney, as well as augmented expression of glycolytic enzymes such as hexokinase 2 [57,58]. These changes were present early in the de-differentiated tubules but persisted with evidence of hypoxia in atrophic tubules at later stages. Thus, glycolytic metabolism exists in persistently injured de-differentiated tubules. However, it is unclear whether glycolysis contributes mechanistically to failed tubule recovery or just reflects ongoing injury.

There are conflicting studies about the role of glycolysis in protecting versus exacerbating AKI. A recent study suggests that blocking glycolysis through inhibitory S-nitrosylation of pyruvate kinase M2 (PKM2), an enzyme that catalyzes pyruvate generation from phosphoenolpyruvate, protected against IRI [59]. The mechanism was thought to be increased flux through the pentose phosphate pathway (PPP), which increases NADPH generation, leading to enhanced glutathione levels and antioxidant enzymes [59]. Thus, by diverting glucose from glycolysis and energy generation towards the PPP, inhibition of PKM2 may protect kidneys by reducing oxidative stress. By contrast, other studies suggest that glycolysis is transiently inhibited in AKI and reversing this inhibition (i.e., promoting glycolysis) protects tubular cells from ischemic injury. Both Poly(ADP-ribose) polymerase-1 (PARP-1) and Tp53-induced glycolysis and apoptosis regulator (TIGAR) were upregulated early after IRI and shown to reduce glycolysis through inhibitory actions on glyceraldehyde-3-phosphate dehydrogenase (GAPDH) and phosphofructokinase (PFK)-1, respectively [60,61]. Blocking PARP-1 decreased tubular cell death in a hypoxia/reperfusion in vitro model, though PARPs mediate many different effects that may be independent of glycolysis. TIGAR expression reduces glycolysis in favor of the PPP and NADPH generation, similar to the effect of blocking PKM2 mentioned above. Interestingly, TIGAR expression augmented the PPP and increased NADPH in mild ischemic injury but not in severe IRI, and TIGAR inhibition protected mice 1 day after severe AKI [61]. Thus, the relative benefit of glycolysis and ATP generation versus PPP and antioxidant function may depend upon the severity of AKI. Alternatively, it may be that TIGAR is unable to augment the PPP in severe AKI and more effective approaches (e.g., PKM2 blockade) would restore the beneficial effect of glycolysis inhibition.

### 4.2. AKI and Glucose Oxidation

The role of renal tubular glucose oxidation in AKI has not been investigated as much as glycolysis. Glucose oxidation is facilitated by pyruvate dehydrogenase (PDH), which converts pyruvate into acetyl CoA, which enters the TCA cycle to form citrate. PDH can be inhibited by phosphorylation at sites S232, S293, and S300 by pyruvate dehydrogenase kinases (PDKs). Hypoxia blocks PDH through the induction of PDKs [62,63]. Renal tubules had increased inhibitory phosphorylation of PDH E1α subunit at 7 days after IRI, and this persisted at 14 days accompanied with atrophic tubules, increased glycolytic enzyme expression, and lactate accumulation [57]. This suggests that increased pyruvate from glucose metabolism in hypoxic injured tubules is producing lactate and not entering the TCA cycle. Cisplatin, a chemotherapeutic agent often limited in use by nephrotoxicity, also increased PDH phosphorylation concurrent with reduced kidney function [64]. Administration of dichloroacetate (DCA), a PDK inhibitor, ameliorated cisplatin-induced renal injury, reduced tubular apoptosis, and prevented the inhibitory PDH phosphorylation [64]. Thus, efforts to augment pyruvate entry to the TCA cycle, or glucose oxidation, may be renoprotective. However, DCA also increased peroxisome proliferator-activated receptor-α (PPAR-α), a regulator of FAO, so the beneficial effects could have been due to these metabolic changes as well.

### 4.3. AKI, Mitochondrial Injury, and Fatty Acid Oxidation

Mitochondrial injury is an important feature of AKI that is closely linked to metabolism. The final step in generating energy from fatty acids involves the mitochondrial ETC and regeneration of NAD+ from its reduced form, NADH. Thus, impaired ETC function in injured mitochondria would reduce NAD+, which is necessary for glycolysis and continued production of ATP from fatty acid or glucose oxidation. Animal models of AKI demonstrate structural and functional mitochondrial injury as reflected by swelling and fragmentation, as well as loss of ETC proteins and a decrease in ATP production [65,66]. Several factors contribute to AKI-induced mitochondrial dysfunction. Hypoxia, a common feature of AKI, leads to increased reactive oxygen species (ROS) accumulation, which can inhibit ETC enzymes [67]. The balance between mitochondrial fusion and fission is disrupted in AKI, leading to mitochondrial fragmentation that can sensitize cells to apoptosis [68]. Additionally, septic models of AKI were shown to have reduced expression of PPARγ coactivator-1α (PGC-1α), an inducer of mitochondrial biogenesis [69]. This PGC-1α suppression was associated with a similar degree of reduced renal function and increased mitochondrial injury. Genetic deletion of PGC-1α in renal tubules exacerbated endotoxin-induced AKI, while overexpression of PGC-1α protected against ischemic renal injury [69,70]. In summary, the hypoxic environment of AKI and cellular responses to these insults result in impaired mitochondrial function. Furthermore, restoring mitochondrial biogenesis through PGC-1α may improve response to AKI.

Mitochondria are necessary for the generation of ATP through fatty acid metabolism, so AKI-induced mitochondrial dysfunction contributes to impaired FAO. PGC-1α activity may augment cellular respiration as proximal tubules that overexpressed PGC-1α in vitro had attenuated TNF-α-induced suppression of basal respiration [69]. As mentioned above, levels of NAD+, critical to FAO, are reduced in AKI. Increased NAD+ consumption through tubular PARPs in rodent ischemic injury contributes to a reduced NAD+:NADH [71]. In addition, metabolomics from the urine of IRI-injured mice revealed impaired expression of enzymes involved in NAD+ biosynthesis and NAD+ levels [70]. However, overexpression of PGC-1α induced NAD+ biosynthesis and rescued the NAD+ levels, suggesting that impaired PGC-1α in AKI may promote further injury by reducing NAD+ levels [70]. Moreover, contributing to impaired fatty acid metabolism after IRI is the reduced activity of CPT1, the rate limiting enzyme for FAO [72]. Augmenting CPT1, using the synthetic compound C75, a fatty acid synthase inhibitor, ameliorated the renal injury in rodents [72]. Data with inhibitors need confirmation with genetic approaches, but these data suggest that impaired FAO may contribute to, rather than just reflect, renal injury.

Recent studies suggest that peroxisomes may also play a role in the renal response to AKI, particularly ischemic AKI. Peroxisomes preferentially oxidize VLCFA, a process that generates hydrogen peroxide (H_2_O_2_) and necessitates large quantities of catalase for H_2_O_2_ metabolism. Thus, peroxisomes may be important in supporting mitochondrial FAO as well as scavenging reactive oxygen species (ROS), both of which are dysregulated in AKI. Peroxisomal FAO, measured by oxidation of the VLCFA lignoceric acid, was reduced proportional to the ischemic time [73]. In both ischemic and cisplatin-induce AKI, deleting the deacetylase sirtuin 5, found to localize to peroxisomes, was protective and decreased mitochondrial but increased peroxisomal FAO [74]. In the cisplatin model of injury, treatment with fibrate, a PPAR-α ligand, reduced renal injury and augmented peroxisomal protein expression [75]. Though these data support a protective role for peroxisomes in AKI, further studies that more directly affect peroxisomal function are necessary to better define whether augmenting peroxisomal FAO protects in AKI.

Taken together, these data suggest that the acutely injured kidney has suppressed fatty acid and glucose oxidation and reduced mitochondrial function with an increase in glycolysis leading to lactic acid (Figure 3). Efforts to increase mitochondrial biogenesis and function or promote fatty acid and glucose oxidation in renal tubules may ameliorate tubular injury and kidney function.

## 5. Renal Tubular Metabolism in CKD

### 5.1. CKD and Fatty Acid Oxidation

Metabolism is significantly dysregulated in human kidneys with CKD. Unbiased transcriptomics of microdissected tubulointerstitial samples from patients with diabetic and hypertensive CKD showed reduced expression of genes related to metabolism of fatty acids, glucose, and amino acids [76]. Although all metabolic pathways were affected, expression of enzymes related to fatty acid metabolism were particularly downregulated. More specifically, gene expression of Cpt1a and Ppara, key regulators of FAO, were reduced in both human and murine models of CKD [76]. In another study, CPT1A levels in human tubules dropped with lower estimated glomerular filtration rates (eGFR), an indication of renal function, and higher rates of fibrosis [77]. Patients from another CKD cohort had increasing accumulation of short- and medium-chain acyl-carnitines with decreasing eGFR, though no change in long-chain acyl-carnitines, which are transported by CPT1a [77]. Blockade of FAO in renal tubules in vitro, using either etomoxir or ranolazine, led to higher levels of cell death and dedifferentiation [76]. Dedifferentiated renal tubule cells contribute to the progression of tubulointerstitial fibrosis [78,79]. The mechanisms whereby reduced FAO leads to tubular dedifferentiation are not entirely clear, but knockout of Cpt1a in cultured endothelial cells led to dedifferentiation through a Smad7/TGF-β-dependent pathway [80].

AKI causes mitochondrial injury, as well as reduced FAO, and, as mentioned above, efforts to reduce mitochondrial injury or optimize fatty acid metabolism improved the response to AKI. There is growing evidence that augmenting mitochondrial biogenesis and/or FAO may also be beneficial in the context of CKD. Genetic overexpression of Ppargc1a, a strong inducer of Cpt1a, specifically in renal tubules reduced tubular apoptosis induced by folic acid [76]. Pharmacologic interventions were used to activate PPARα or block CPT1, using fenofibrate and etomoxir, respectively, in the unilateral ureteral obstruction (UUO) model in which a ureter is ligated, leading to rapid development of kidney fibrosis over 5–7 days due to backpressure and inflammation [76]. While there are recently reported off-target effects of etomoxir [81], these data indicate that augmenting FAO in renal tubules through Cpt1a or Ppara improves the response to renal injury.

More recently, genetic overexpression of Cpt1a in the renal tubules protected against three murine models of CKD: folic acid nephropathy, UUO, and adenine-induced nephrotoxicity [77]. Folic acid nephropathy involves a single injection of folic acid that causes crystal formation in tubules, leading to tubulointerstitial fibrosis. Adenine, administered through the diet over several weeks, also induces crystal deposition in tubules and fibrosis. In addition to rescuing FAO in ex vivo tissue measured by [^14^C]palmitate studies, Cpt1a overexpression improved mitochondrial morphology and ATP generation after folic acid nephropathy [77]. These elegant studies strongly suggest that suppressed FAO plays a pathogenic role in the progression of tubulointerstitial fibrosis.

### 5.2. CKD Models and Fatty Acid Oxidation

No rodent model of CKD perfectly recapitulates human CKD. Many commonly used CKD models have an initial AKI component (e.g., IRI, folic acid nephropathy, and aristolochic acid nephropathy), and the effect of FAO on AKI may then dictate the progression to CKD. Many of the studies related to FAO and CKD have used the folic acid model [76,77], raising the question of how much of the protective effect of FAO was due to its role in the acute phase of injury. One study did show that the protective effect of Cpt1a overexpression was maintained even when recombination was induced after injury, suggesting that FAO may play a role after the acute injury [77]. However, doxycycline was given to the tet-inducible Cpt1a overexpressing mice just one day after folic acid injections, raising the question of whether Cpt1a did modulate the acute phase of injury. The UUO model, a classic model of tubulointerstitial fibrosis, is more suited to induction of rapidly progressive fibrosis than assessing epithelial injury and repair. Published studies are encouraging that FAO augmentation may be protective in CKD, but future studies should also examine the role of FAO in more gradually progressive models that reflect hypertensive nephrosclerosis and/or diabetic nephropathy, the two leading causes of end-stage kidney disease.

### 5.3. Fatty Acid Oxidation and Tubulointerstitial Fibrosis

There are several mechanisms whereby increasing FAO may reduce tubulointerstitial fibrosis. Lipid accumulation in the kidney, whether by reduced metabolism, increased uptake, or increased synthesis, is a feature of human CKD [82,83,84]. In addition to the downregulation of lipid metabolism, expression of the fatty acid receptor CD36 is upregulated in CKD [82]. Mice with CD36 inhibited either genetically or pharmacologically were protected against a model of hypertensive CKD [85]. Several groups have proposed that excess lipids in the kidney promote CKD progression through enhanced inflammation, oxidative stress, and endoplasmic reticulum (ER) stress [86,87]. Consistent with this, mice lacking CD36 placed on a high-fat diet were also protected from UUO-induced kidney injury with suppressed pathways that mediate inflammation (e.g., NF-κB) and oxidative stress [88]. Mice lacking proximal tubular carnitine acetyltransferase, the enzyme that exports excess acyl CoA products out of the mitochondria, spontaneously developed increased apoptosis, fibrosis, and oxidative stress [89]. These findings were accelerated by a high-fat diet and associated with impaired mitochondrial function [89]. However, overexpression of CD36, while increasing the accumulation of fatty acids in the renal tubules, did not significantly impact fibrosis induced by either streptozotocin-induced injury, a model of type I diabetes, or folic acid nephropathy [76]. There is strong evidence to support the roles of CD36 and lipotoxicity in progression of CKD, but the exact contribution likely depends upon the injury model and other modifying factors (e.g., diet).

Another putative mechanism by which CPT1a and FAO may reduce fibrosis is through TGF-β signaling. As mentioned above, endothelial cells lacking Cpt1a had increased de-differentiation through a TGF-β/Smad7 pathway [80]. Similarly, primary kidney tubule cells from Cpt1a overexpressing mice had attenuated de-differentiation in response to TGF-β1 [77]. These in vitro data need to be validated in vivo, and the question remains of whether these effects of CPT1a are FAO-dependent or not.

Reduced FAO may also promote tubular atrophy, a component of tubulointerstitial fibrosis, through impaired ATP generation. Etomoxir-dependent inhibition of FAO in tubular cells led to suppressed ATP generation and increased apoptosis in vitro [76]. This suggests that reduced FAO may lead to cell death through impaired ATP production. Augmenting Cpt1a improved mitochondrial morphology by EM in renal tubules [77], suggesting that increasing FAO may also be protective through improving mitochondrial function. Thus, FAO may reduce tubular injury and tubulointerstitial progression by reducing oxidative stress and inflammation from lipotoxicity, reducing tubular TGF-β signaling, and increasing tubular survival through improved ATP production and mitochondrial function. However, the connection between FAO and tubulointerstitial fibrosis in CKD requires further study to confirm these potential mechanisms.

### 5.4. CKD and Anaerobic Glycolysis

In both human and animal models, enzymes related to anaerobic glycolysis are increased in CKD [57,76,90]. This is unsurprising as mitochondrial injury is an integral part of CKD, and glycolysis leading to lactic acid production can generate ATP without the need for functioning mitochondria. The role of glycolysis in the progression of CKD is less well defined. In the UUO model, inhibition of glycolysis through 2-deoxyglucose, injection of lentiviral PKM2 RNAi, or treatment with shikonin reduced fibrosis, as well as myofibroblast activation [90]. The mechanism was thought to be mediated by renal fibroblasts (NRK-49F), which responded to PKM2 treatment with increased lactate content and myofibroblast activation (increased fibronectin expression, α-SMA, and proliferation marker PCNA) [90]. Another group also used shikonin in UUO-injured mice and reported reduced fibrosis and tubular apoptosis though effects on matrix production in vitro were only seen on fibroblasts and not epithelial cells [91]. These studies suggest that blocking glycolysis protects against fibrosis in the UUO model, though the beneficial effects appears to be mediated primarily through fibroblasts rather than tubular cells.

Other studies suggest that anaerobic glycolysis may not be deleterious in CKD. In contrast to the protective effect of blocking PKM in AKI, the PKM activator TEPP-46 reduced fibronectin and other matrix-associated gene expression in tubules of streptozotocin-treated mice [92]. TEPP-46 also ameliorated podocyte injury and basement membrane thickness, so it is possible that the tubular protection may be due to reduced glomerular injury and proteinuria rather than direct effects on tubular glycolysis [92]. Another study used a novel genetic approach to interrogate the role of glycolysis in renal injury. Transgenic mice containing an inactivating point mutation in a key glycolytic enzyme, 6-phosphofructo-2 kinase/fructose-2,6-bisphosphatase (PFKFB2), had reduced glycolytic capacity [93]. These transgenic mice with decreased glycolysis were not protected against UUO or folic acid–induced fibrosis, and fibrosis was exacerbated in the transgenic mice injured by UUO [93]. The UUO model targets the distal tubules, though not exclusively, which tend to rely more on glycolysis for metabolism, thus a more proximal tubule-specific injury could yield different results. Alternatively, it may be that some glycolytic capacity may be important for injury response. It is possible that if the injury is severe enough, glycolysis might be necessary to generate energy temporarily until repair can occur. Future studies should address whether blocking glycolytic capacity after the initial kidney insult might reduce tubulointerstitial fibrosis progression.

### 5.5. Unanswered Questions in Injured Tubular Metabolism

In summary, the metabolically active kidney tubules support their metabolism with FAO and glycolysis in the proximal tubules and distal tubule segments, respectively. After AKI and CKD, mitochondrial injury and reduced nutrient availability significantly change tubular metabolism. Elegant studies have recently provided insight into ways to mitigate tubular injury through changes in metabolic or mitochondrial function, suggesting the importance of metabolism in kidney injury and fibrosis progression. However, additional studies are needed to better understand how tubular metabolism can be modulated to improve the response to renal injury. Recent data strongly support a protective effect of augmenting FAO in CKD, even when upregulated after the initial injury [76,77]. These studies have been largely done in rodent models in which tubule injury is either induced by crystal formation (folic acid nephropathy and adenine-induced nephropathy) or obstruction (UUO). Although many features of tubulointerstitial progression are similar regardless of the initial insult, further studies in rodent models that more closely resemble hypertensive or diabetic kidney disease are important to confirm these important findings. The protective effect of FAO is also suggested in AKI, though most studies have indirectly manipulated CPT1a expression through modulation of other genes. Increasing FAO augments oxygen consumption and might theoretically sensitize tubules to ischemic injury, so further studies that directly modulate CPT1a or FAO in AKI are warranted.

In the injured kidney, glycolysis increases with increasing severity of injury. However, it remains unclear whether glycolysis is an adaptive metabolic strategy to generate ATP in the context of mitochondrial energy and hypoxia or maladaptive response. There are data suggesting that glycolysis may activate fibroblasts into matrix-producing myofibroblasts. In renal tubules, there may be a tradeoff between the antioxidant benefits of blocking glycolysis and increasing the PPP versus reduction in ATP generation. This balance may depend not only upon severity of renal injury but also duration and requires further investigation. Finally, the role of glucose oxidation in AKI and CKD is largely unexplored except for a study supporting a protective role for glucose oxidation in cisplatin-induced AKI [64]. Metabolic flexibility between fatty acid and glucose oxidation is important in cardiac injury [94,95,96] and may have a protective role in the kidney. An improved understanding about which metabolic changes in renal tubular injury are protective versus maladaptive will hopefully lead to new therapeutic avenues to treat AKI and CKD.
